# Fortification of Ogi with Whey Increases Essential Amino Acids Content of Fortified Product

**DOI:** 10.1155/2017/7450845

**Published:** 2017-08-07

**Authors:** J. O. Omole, O. M. Ighodaro, O. Durosinolorun

**Affiliations:** Department of Biochemistry, Lead City University, Ibadan, Oyo State, Nigeria

## Abstract

The inability of humans to synthesize essential amino acids (EAA) necessitates the need to increase the levels of these nutrient molecules in certain foods in which they are deficient. Maize ogi is a typical food product for both infants and adults in Africa, but with poor EAA content. This study therefore sought to assess the possibility of increasing the EAA content in maize ogi by processing it with cheese whey instead of water. Maize ogi and whey-fortified ogi were prepared by the usual procedure of grain soaking, milling, and drying. Samples from both treatments were subjected to proximate composition and amino acid profile analyses using Waters 616/626 LC (HPLC) instrument. L-lysine, L-trytophan, and L-methionine contents in maize ogi remarkably increased from 0.52, 0.15, and 0.90 mg/100 gm sample, respectively, to 0.90, 240, and 1.320 mg/100 gm sample in whey-fortified ogi. There were also significant increases in other EAA contents of whey-fortified ogi relative to its counterpart (normal maize ogi). The sum increase in EAA contents (9,405 mg) correlates with the increase in protein (1 gm) per gram sample. This study demonstrates that cheese whey increases EAA content in maize ogi and suggests that whey-fortified maize ogi may be a preferred alternative to water processed maize ogi.

## 1. Introduction

Essential amino acid content determines the nutritional and functional quality of proteins which in turn play several important roles in body physiology and development. Humans and some farm animals lack the cellular proficiency to synthesize these amino acids (essential), thus, necessitating the need to increase the levels of these nutrient molecules in certain foods in which they are deficient. Ogi is a traditionally fermented food made from cereals including maize, millet, and sorghum and commonly consumed by both infants and adults in Africa, particularly West African countries including Nigeria, Ghana, and Ivory Coast [1–4].

Maize is commonly used for ogi production and thus imparts on the nutritional quality of the ogi made from it. Maize ogi, though widely consumed, has poor nutritional quality due to deficiency in some essential amino acids including L-lysine, L-tryptophan, and L-methionine in maize [4, 5]. This has been attributed to a relatively large proportion of prolamins which are essentially devoid of lysine and tryptophan [5]. In fact normal maize varieties of protein contain on an average about 2% lysine which is less than one-half of the concentration recommended for human nutrition by the Food and Agriculture Organization (FAO) [6].

Breeding for improved protein quality in maize led to the discovery of a mutant gene that changes protein composition and increases lysine content of maize endosperm, and this is opaque-2 maize [7]. Besides, various research efforts have taken place all aimed at improving the nutrient quality of maize ogi, among which are fortification of ogi with fermented or unfermented legumes [2, 8, 9], addition of pawpaw slurry at varying levels of substitution [9], and fortification with okra seeds [10]. Unfortunately, all these research efforts only address the protein content of maize ogi rather than the deficiency in those limiting amino acids. It is important to state that L-methionine is the third limiting amino acid in normal maize after L-lysine and L-tryptophan and it is the first limiting amino acid in legumes. Thus, in a complete diet of maize and legume, methionine can be nutritionally limiting [11].

The use of whey, which is a green yellow translucent watery portion of milk remaining after milk coagulation and removal of the curd or cheese, as an ingredient in food processing is well documented [12, 13]. Whey contains high quality and nutritious dairy proteins like alpha-lactoglobulins, beta-lactoglobulins, bovine serum albumin, and immunoglobulins which are good for healthy living because of their richness in essential amino acids, especially the branched-chain amino acids which include leucine, isoleucine, and valine [14]. It was on this basis that maize was processed with whey in the present study with a view to producing a whey-maize ogi that would not be limiting in any essential amino acids.

## 2. Materials and Methods

### 2.1. Preparation of Maize Ogi with Distilled Water

Normal maize for the production of maize ogi was purchased from a local market (Bodija market) in Ibadan, Nigeria. The procedure of Akingbala et al. [1] with slight modification was used for the production of maize ogi. The flow chart for the production is shown in Table 1. Maize grains (1.5 kg) were soaked in distilled water for 72 h to allow fermentation to take place. The grains were removed, drained, and wet milled at the university food processing workshop mill using a locally fabricated wet and dry mill. The slurry obtained was sieved using affine sieve of diameter 300–400 micrometers. The suspension obtained was left to stand for 24 h to allow the ogi to settle. The suspension was decanted and the ogi collected and dried in a tray dryer for 48 hours at 80°C. The dry form was ground to powder and then packed in nylon for safe storage until being required for analysis.

### 2.2. Preparation of Whey-Maize Ogi

Whey for this purpose was purchased from Fulani cheese makers located at toll gate area, Ibadan, Nigeria. Maize grains (1.5 kg) were cleaned and washed with water before being soaked in whey for 72 h. After steeping, the maize grains were removed and drained. The steeped grains were milled and the slurry obtained was sieved with a fine sieve of diameter 300–400 micrometers. The filtrate was left to stand for 24 h to allow the ogi to settle. The suspension was decanted and the solid remaining was collected and dried in a tray dryer for 48 h at 80°C. The powdered whey-maize ogi was similarly stored in nylon and kept in safe storage until being required for analysis.

## 3. Proximate Composition Analysis

Normal maize ogi and whey-maize ogi samples were subjected to proximate composition analysis for moisture, crude fat, crude protein, ash, and fibre contents using standard procedures of Official Methods of Analysis (AOAC) [15]. The carbohydrate contents of the samples were obtained by difference.

### 3.1. Amino Acid Analysis

Amino acids were determined by High Performance Liquid Chromatography (HPLC) by the method of AOAC [15]. Briefly, 0.5 g of ground sample was hydrolysed in sterile furnace hydrolysis tube using 10.5 N HCL with a small quantity of phenol (HCL Vapour) between 20 and 23 h at 108°C. The hydrolysed samples were then dissolved in ultrapure water (HPLC grade) containing EDTA to chelate any metal present in the sample and the samples were thereafter stored in HPLC Amino Acid Analyzer bottles for further analytical operations.

### 3.2. Derivatisation of Hydrolysed Samples

The hydrolysed samples were derivatised automatically on the Waters 616/626 HPLC by reacting the five amino acids under basic situations with phenylisothiocyanate (PITC) to obtain phenylthiocarbamyl (PTC) amino acid derivatives.

### 3.3. Preparation of Standard Amino Acids

Standard amino acids (0.0, 0.5, 1.0, 1.5, and 2.0 umol) prepared from pierce reference standards H (1000 umol) were used to generate a calibration curve that was used to determine the amino acids contents of the samples. The standards were also derivatised. After the derivatisation, a methanol solution (1.5 N) containing the PTC-amino acid was transferred to a narrow bore Waters 616/626 HPLC system for separation.

### 3.4. Separation and Quantification

The separation and the quantification of the PTC-amino acids were done on a C18-reversed phase silica gel column, and PTC chromaphone was automatically and digitally detected at the wavelength of 254 nm. The buffer system used for separation was 140 umol sodium acetate (pH 5.5) as buffer A and 80% acetonitrile as buffer B. The program was run using a gradient of buffer A and buffer B concentration and ending with a 55% buffer B concentration at the end of the gradient elution. The intensity of the chromatographic peaks areas was automatically and digitally identified and quantified using a dionex chromeleone data analysis system attached to the Waters 616/626 HPLC system

### 3.5. Statistical Analysis

Data were comparatively analyzed using independent sample *t*-test

## 4. Results and Discussion

The dietary requirement of high protein and low carbohydrate diets by consumers for obvious reasons, particularly for nutritional and health benefits, and it is necessitating the need for protein-fortified food products. Ogi is a popular meal among adults and commonly used as a weaning food for babies. It is however low in nutritional quality due to lack of essential amino acids. In this study, we hoped that fortification of ogi with whey will improve its protein quality and invariably enhanced its nutritional quality.

A comparison of the proximate compositions of normal maize ogi and whey-maize ogi showed an increase in protein content (9.53%), fat content (0.5%) and ash content (0.57%) of whey-maize ogi as shown in Table 1. Except for protein, the increases in other nutrients were however not significant (*p* < 0.05). Whey contains high quality and nutritious dairy proteins like alpha-lactoglobulins, beta-lactoglobulins, bovine serum albumin, and immunoglobulins and is good for healthy living due to its richness in the essential amino acids especially the branched-chain amino acids which include leucine, isoleucine, and valine [16]. It is also rich in minerals. Thus, the observed increase in protein and ash content of the whey-fortified maize ogi is arguably due to nutrients contribution from whey

Table 2 shows a comparison of the essential amino acids content of normal maize ogi and whey-maize ogi. There was a substantial and highly significant (*p* < 0.05) increase in the essential amino acids content of whey-maize ogi. The total weight of essential amino acids found in whey-maize ogi was 9,625 mg while the total essential amino acid in normal maize ogi was 226.13 mg. Thus, there has been an increase of 9,399 mg as a result of fortification with whey, which corresponds to the 1.02 gm increase in crude protein content noted in this study. This study again confirms the unique richness of whey in all the essential amino acids and its suitability as an ingredient in food processing by providing a combination of animal protein with plant protein.

## 5. Conclusion

This study demonstrates that cheese whey increases essential amino acid content in maize ogi and suggests that whey-fortified maize ogi may be a preferred alternative to normal maize ogi. More so, the data obtained may provide useful information in the formulation of infant feeding formula rich in quality protein and minerals, which are essential for child growth and development.

## Figures and Tables

**Table 1:** Proximate composition of normal maize ogi and whey-maize ogi.

Nutrient	Normal maize ogi (%)	Whey-maize ogi (%)
Moisture	6.00 ± 0.03	5.74 ± 0.35
Protein	10.70 ± 0.28	^*∗*^11.72 ± 0.18
Fat	7.60 ± 0	8.10 ± 2.90
Ash	1.00 ± 0	1.37 ± 0.35
Crude fibre	0.05 ± 0	0.05 ± 0
Carbohydrate	74.60	72.82

Values are means of 3 determinations with standard deviations. *∗* = significant at *p* < 0.05.

**Table 2:** Essential amino acids content of normal maize ogi and whey-maize ogi.

Amino acid	Normal maize Ogi	Whey-maize Ogi
Lysine	0.53 ± 0.02	^*∗*^90.00 ± 0
Tryptophan	0.15 ± 0	^*∗*^240.00 ± 0
Methionine	0.90 ± 0.01	^*∗*^1,323.00 ± 0
Leucine	1.20 ± 0.02	^*∗*^1,240.00 ± 0
Iso-Leucine	2.53 ± 0	^*∗*^830.00 ± 0
Valine	0.84 ± 0.01	^*∗*^83.30 ± 0.01
Threonine	216.00 ± 0.02	^*∗*^4,780.00 ± 0
Histidine	0.281 ± 0.01	^*∗*^520.0 ± 0
Phenylalanine	3.50 ± 0	^*∗*^625.0 ± 0.01
Total	226.13	9,631

Values are means of 3 determinations with standard deviations. Unit = mg/100 g prod. *∗* = significant at *p* < 0.05.
